# 271. Epidemiology of ESBL *Klebsiella pneumoniae* Clonal Group 307 in Stony Brook, NY

**DOI:** 10.1093/ofid/ofad500.343

**Published:** 2023-11-27

**Authors:** Jessica Glicksberg, Monirul I Sajib, Sanjana Sankaran, Marissa Lindner, Jonathan Mui, Camila Boniche-Alfaro, Bettina F Fries

**Affiliations:** Stony Brook Medical Center, Stony Brook, New York; Stony Brook University Hospital, Stony Brook, New York; Stony Brook University, Stony Brook, New York; Stony Brook University, Stony Brook, New York; Stony Brook University, Stony Brook, New York; Stony Brook University, Stony Brook, New York; Renaissance School of Medicine at Stony Brook University, Stony Brook, NY

## Abstract

**Background:**

According to the most recent CDC threat report infections caused by ESBL and carbapenem-resistant *Klebsiella pneumoniae* (CR*Kp*) remain a persistent challenge. In the US dissemination of CR*Kp* has been largely attributed to clonal group (CG) 258 with recent evidence that CG307, a dominant Klebsiella Extended Spectrum Beta-Lactamase (ESBL-*Kp*) clone is also emerging as CR*Kp*. CG307 strains uniformly express a wzi-173 type capsular polysaccharide (CPS) whereas other Klebsiella strains including those of the CG258 express a wide variety of wzi-type CPS.

A paper from Houston, TX provided evidence of divergent evolution between CG258 and CG307. CG258 evolves upfront as carbapenem resistant whereas CG307 may initially emerge as ESBL. CR-*Kp* that belong to the CG307 have also been identified in SBU. Our objective was to determine if among ESBL-*Kp* CG307 is a common clone and to characterize the demographics of the infected patient population.

**Methods:**

Molecular typing of the wzi gene alleles of 78 sequentially collected ESBL-*Kp* was performed. Demographic, microbiological and clinical data were extracted from the medical record.

**Results:**

Wzi typing indicated that 43% of ESBL*Kp* strains express a wzi173 type CPS indicating they belong to CG307 the remainder of ESBL strains expressed variable wzi-types. Preliminary data analysis showed that wzi173 expressing ESBL strains were predominantly derived from urine. The median age of patients was 64 years old with male predominance (60.8% males). The average length of stay is 41.6 days. Among these patients, 30.4% required ICU admission. Sepsis criteria were met in 56.5% of these patients within 2 days of positive culture. The mortality rate was 21.7%.

**Figure:**
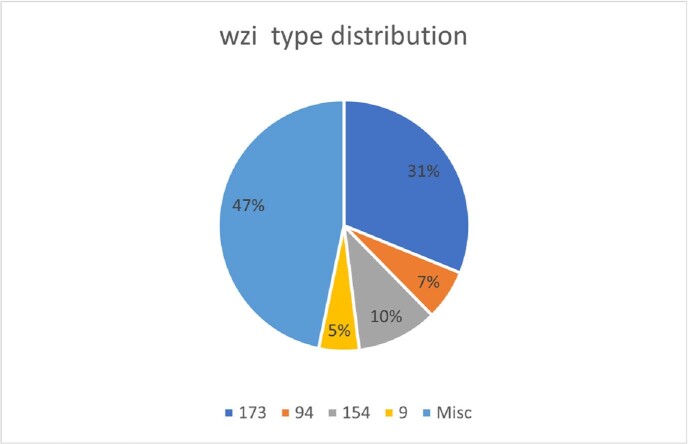

**Figure:**
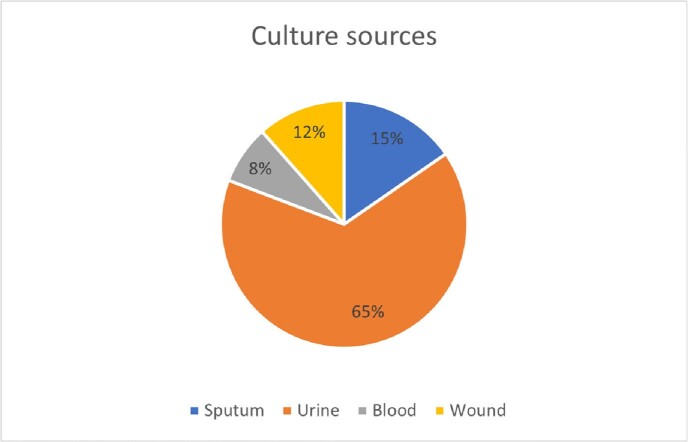

**Figure:**
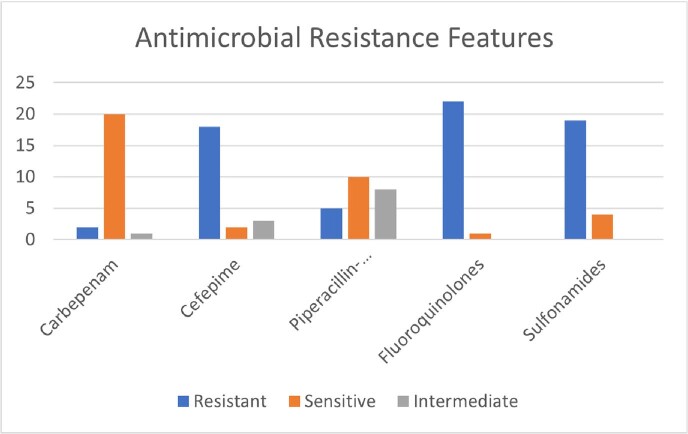

**Conclusion:**

Our data provides evidence that CG307 is the most dominant ESBL *Kp* clone in hospitalized patients at Stony Brook University. Although the majority of cultures were from urinary source, ICU admission rates and mortality rates were also elevated. These data raise concern that high prevalence of ESBL CG307 in hospitalized patients may ultimately lead to emergence of carbapenem resistant CG307 strains especially if they emerge in patients who are treated with broad spectrum antibiotics.

**Disclosures:**

**All Authors**: No reported disclosures

